# Forensic Age Estimation: A Multifactorial Approach in a Retrospective Population Study

**DOI:** 10.3390/diagnostics13122029

**Published:** 2023-06-12

**Authors:** Monika Bjelopavlovic, Sebastian R. Reder, Isabel Fritzen, Marc A. Brockmann, Jochen Hardt, Katja Petrowski

**Affiliations:** 1 Department of Prosthetic Dentistry, University Medical Center, Johannes Gutenberg University Mainz, Augustusplatz 2, 55131 Mainz, Germany; isabel.fritzen@unimedizin-mainz.de; 2 Department of Neuroradiology, University Medical Center, Johannes Gutenberg University Mainz, 55131 Mainz, Germany; sebastianreder91@gmail.com (S.R.R.);; 3 Department of Medical Psychology and Medical Sociology, University Medical Center, Johannes Gutenberg University Mainz, Duesbergweg 6, 55131 Mainz, Germany; hardt@uni-mainz.de (J.H.); kpetrows@uni-mainz.de (K.P.)

**Keywords:** forensic sciences, forensic dentistry, dental age estimation, Demirjian staging, wisdom teeth, clavicle, forensic age estimation

## Abstract

**Objectives**: The objective of this study was to evaluate the accuracy of forensic age estimation in a German population by combining clavicle and wisdom teeth assessments based on cone beam computed tomography (CT) data. The study aimed to determine the reliability of this approach in predicting biological age. **Material and Methods:** A total of 161 CT data sets from 120 males and 41 females with known exact ages were evaluated by three raters. The clavicle was assessed according to stages 1–5 (including substages 2a–c and 3a–c), and the Demirjian stage’s classification method was used for the wisdom teeth. Inter-class correlation (ICC) was calculated to assess the agreement among the three raters. Additionally, ordinary least square regressions were performed to predict chronological age using the clavicle or one of the four teeth. Finally, age prediction models using multiple indicators were developed. **Results:** The ICCs ranged from 0.82 for the clavicle to 0.86 and 0.88 for the wisdom teeth. Linear estimation tended to overestimate chronological age, especially in subjects over 18 years old. The clavicle showed the strongest overestimation. Combining age estimation from the clavicle with the upper and lower wisdom teeth improved the predictive power, resulting in a 14% and 15% increase in R² for the upper and lower wisdom teeth, respectively. Adding more than one tooth to the prediction did not improve the predictive power (all ΔR² < 1%). **Conclusions:** Age estimation using CT can be significantly improved by combining information from the analysis of wisdom teeth with age estimation based on the clavicle.

## 1. Introduction

Age determination based on biological structures has become a standardized procedure to determine the most probable age of an individual based on different developmental stages. X-ray based analysis of wisdom teeth and skeletal development of the hand as well as of the clavicle are well established methods [1,2]. The indications for age determinations exist within and outside of criminal proceedings and are commissioned by courts, youth welfare offices and district administrations [3,4,5]. Additionally, age determination is often employed in cases involving unidentified corpses and skeletons to aid in identification [6,7]. The legally relevant age limits of 14, 18, and 21 years are of particular interest in this context and may have an impact in criminal proceedings on the overall verdict and, among other things, on the culpability of the defendant. In Germany in particular, the age of criminal responsibility is defined as the age of 14 (§19 StGB) [8]. Attaining the age of 18 establishes legal adulthood, and reaching 21 years applies adult criminal law [9]. Consequently, clear guidelines need to be followed, which are defined nationally in Germany by the AGFAD (working group for forensic age estimation) [5]. Scientifically sound methods and an indication for radiological clarification are required to ensure the most exact approximation of the true biological age. Consequently, the interaction of three expert groups including radiology, dentistry, and forensic medicine is required [10,11].

In the field of radiology, it is common practice to obtain a two-dimensional hand radiograph to determine the age of an individual [5]. However, in cases where hand growth is complete, a thin-slice CT scan of the clavicle can be performed, and staging can be applied using reference tables and standard deviations [5,10]. The medial clavicular epiphysis is the last bone to ossify during development and therefore provides valuable biological information about an individual’s age [12,13]. The original subdivision of ossification into stages 1–5 has been further refined with the addition of subgroups. Substage 3c indicates completion of 19 years of age, and the presence of stage 4 suggests that an individual has completed 21 years of age [14,15]. The clavicle has been identified in the literature as a reliable biological structure for forensic age estimation and can play a supporting role in determining the question of majority [16,17]. It is worth noting that the accuracy of age estimation is not affected by the dose of the CT scan [18]; however, professional experience has been found to be an important factor in this regard [19]. Age estimation combining information from both clavicle and wisdom teeth analyses has been reported in the literature. However, only one study has investigated both structures individually using a single CT dataset [20]. A multifactorial approach is crucial when it comes to AGFAD standards, and it must be population-based to ensure an accurate expert opinion. Overall, the use of a thin-slice CT scan of the clavicle provides a reliable method for determining an individual’s age and can be used in conjunction with other methods for forensic age estimation [5,11,21]. In science, further methods are being investigated that show promising results for certain age groups, such as age diagnosis using the fourth vertebra in a study by Gulsahi et al. They investigated the correlation between age and the fourth cervical vertebra in a Turkish population group including children and young adults and could demonstrate the suitability of this method [22]. Another study by Cameriere et al. also investigated the relationship between age and the fourth vertebra and developed a statistical model based on the ratio of anterior to posterior vertebral height [23].

Dental age diagnosis includes the Demirjian staging method and divides the development of the wisdom teeth into eight (A–H) stages [24,25]. Wisdom teeth exhibit a wide variance in shape, eruption timing, and development, with the most significant changes occurring between the ages of 11 and 24. Due to its extended developmental period, wisdom teeth are ideal for estimating age within the legally relevant limits, particularly for assessing individuals under the age of 21 [26,27]. Teeth have been described in the literature as biological structures that exhibit greater resistance to malnutrition and other epigenetic factors compared to skeletal development [28]. The staging used with corresponding reference tables in expert reports is based on sex-specific population studies [29,30,31,32] with a respective standard deviation and indication of minimum and maximum ages. A slowed wisdom tooth development in a secular context was observed in a Croatian population sample in 2007–2009 compared to the 1977–1979 annual range [33]. In conclusion, dental age diagnosis based on wisdom tooth development is an important tool for estimating age within the legally relevant limits. The Demirjian staging method, sex-specific population studies, and reference tables are used to ensure accurate age estimation. However, due to variations in wisdom tooth development, it is necessary to continuously validate and update existing reference tables and studies to maintain the reliability and accuracy of age estimation methods [34,35,36].

Overall, the presence of wisdom teeth is a fundamental requirement and is not always guaranteed due to possible agenesis. The wisdom teeth are more often not in place when other forms of agenesis are present [37]. In addition, it could be observed in this group that a symmetrical agenesis of the wisdom teeth occurs with a threefold increased probability in the upper as well as in the lower jaw and, overall, an evolutionary “tendency to fewer teeth” can be confirmed [37,38].

In addition to the possible agenesis of wisdom teeth, they may also be missing due to surgical extraction. According to guidelines, wisdom teeth are extracted in young adulthood in case of inflammation, cysts, or lack of space [39,40]. Thus, the indication is patient-specific and does not always involve all four wisdom teeth of an individual at the same time. If all four wisdom teeth are discoverable, the literature indicates greater accuracy in the mandible’s wisdom teeth related to the biological age than in the wisdom teeth of the upper jaw [41,42]. Often the maxillary wisdom teeth are not located in this context due to reduced image quality, and a study by Bjelopavlovic et al. showed that the combination of tooth 38 and another, preferably tooth 48, would show the most accurate results in terms of dental age diagnosis [42]. As a limitation of the generalizability, this study involved the evaluation of wisdom teeth using only 2D radiographs without including other methods such as staging of the clavicle.

In our study, we investigated age estimation using CT datasets that included all four wisdom teeth as well as the clavicles in a patient population with known biological age. Specifically, we aimed to investigate interrater differences in dental age estimation among experts at different levels of expertise. Furthermore, we aimed to determine whether forensic age estimation is more accurate when all five indicators (wisdom teeth 18, 28, 38, 48, and the clavicle) are used simultaneously.

Our study was based on the following hypotheses.

(1) The dental age determination of the three raters differs according to their expertise.
(2) Forensic age estimation is more accurate when all five indicators (wisdom teeth 18, 28, 38, 48 and clavicle) are used.

## 2. Materials and Methods

### 2.1. Subject Group

A total of 161 CT data sets of 120 male and 41 female patients with known age acquired between 2001 and 2022 at the University Medical Center in Mainz were retrospectively evaluated. All CT scans were performed in the context of different medical indications. We followed the ethical standards of the local ethics committee and analyzed our data sets retrospectively. The subjects were between 12 and 25 years of age (mean age 17.6).

### 2.2. Inclusion Criteria

The study population consisted of subjects with a known date of birth who had undergone a CT scan during the specified period. The CT scans included the head and shoulders in the field of view. 

### 2.3. Exclusion Criteria

Subjects who did not have at least two wisdom teeth or those with a lack of visualization of the clavicles were excluded from the study.

### 2.4. Design

Patient age using the clavicle was determined by three raters and categorized according to Wittschieber et al. (stages 1–5 with substages 2a–c and 3 a–c). The patient age according to the wisdom teeth was measured by the same three raters and categorized using stages A to H according to Demirjian. The patient age was finally calculated as follows: date of examination minus date of birth/365.25. Generally, a person was understood to be 18 from the day of their 18th birthday to the day before their 19th birthday. No rounding was applied here. Hence, the accuracy of the chronological age was considered to be exact ±1 day. 

**Figure 1:**
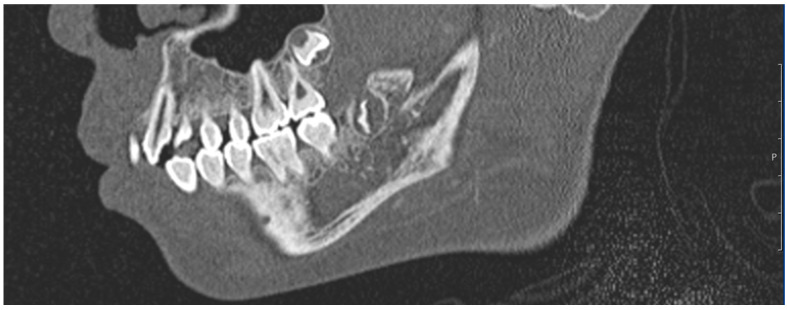
CT image of a 14-year-old subject with wisdom teeth 18 and 48.

**Figure 2:**
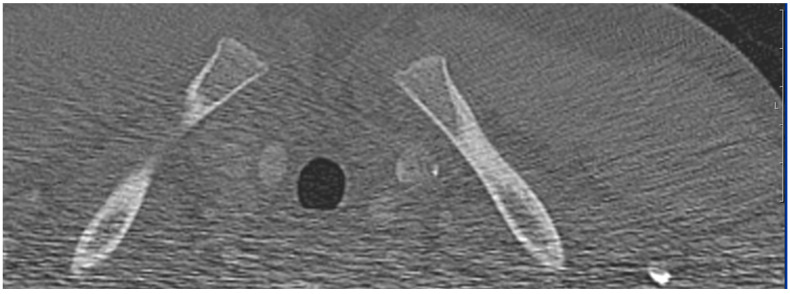
CT image of a 14-year-old subject with the right and left clavicle.

The three readers evaluated the CT datasets independently from each other within the same period of four weeks at the same time of day (between 5:00 and 10:00 p.m.). All CT scans were read using calibrated radiological viewing monitors according to DIN 6868-157. The reporting took place at the Department of Neuroradiology under standardized conditions as defined by the Swiss Society for Diagnostics [43]. All three raters were provided unrestricted access to all features and display modes of the SECTRA^®^ CT software, including the ability to use contrast and magnification functions at their discretion. Every rater reported the radiological stadium of the wisdom teeth (Figure 1) and the clavicle (Figure 2) according to the AGFAD standard. The data were collected separately from all three raters in an Excel table, which only contained a patient code without any information about age. The pseudonymized reader-specific Excel data sets were acquired separately. The chronological age was entered afterwards and before statistical analysis. To be concrete, three tables were created with the chronologically known age, the clavicle stage of the right and left clavicles and the stages of the wisdom teeth. The stages were assigned to the respective age according to the AGFAD standard from the corresponding reference tables.

### 2.5. Statistical Analysis

First, interclass correlations for the three raters were calculated for the clavicle and the four teeth. In this study, ICC was used as an indicator of reliability between the three raters in determining the age of the subjects based on the clavicle and wisdom teeth stages. Second, simple linear ordinary least square regressions were calculated predicting the chronological age by clavicle or one of the four teeth. Finally, age prediction using multiple indicators was performed. Because not every proband had all four wisdom teeth, there are some missing data in the final analyses. The method of multiple imputation via chained equations (MICE) with predictive mean matching was applied to deal with it [44]. In short, the procedure created 100 samples, where the missing data were substituted via a linear prediction with some random error added. The latter was done to address the uncertainty of the substitution. Finally, regression analyses were performed in each sample, and the coefficients were combined using Rubin’ s rules [45]. This procedure leads to more precise and less biased estimates than using a complete case analysis [46]. All analyses were performed using STATA V17 (STATA Corp. STATA V17. 2022). 

## 3. Results

Table 1 gives a description of the sample and reports the ICCs among the three raters as indicators for reliability. ICCs range from 0.82 for the clavicle to 0.86 and 0.88 for the wisdom teeth. The reliability can be said to be good.

**Figure 3:**
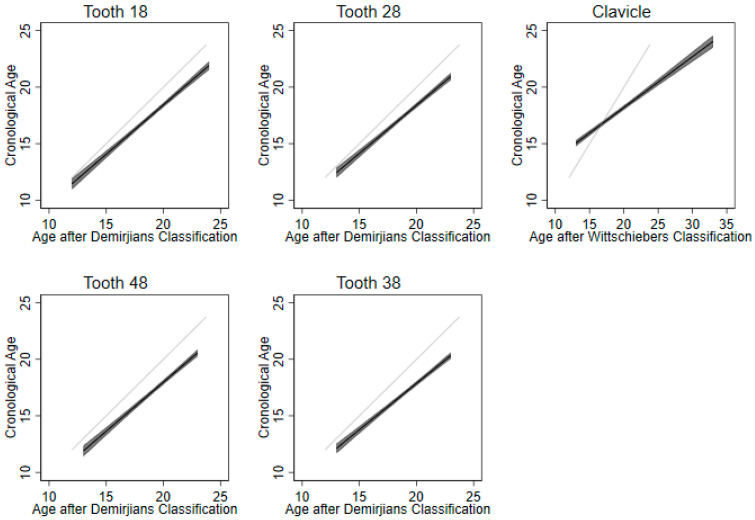
Age estimated by the third molars and clavicula.

Figure 3 and Table 2 and Table 3 address validity by predicting chronological age. When utilizing the clavicle or the mineralization of each tooth individually (tooth 18, 28, 38, 48), R² values are in the range of 0.57–0.65. Wisdom teeth show somewhat higher R² values than the clavicle; correspondingly, the beta of the clavicle (b = 0.45) is smaller than those of the wisdom teeth (0.82 < b < 0.87), and its constant is higher (9.19 vs. <1.44). 

Figure 3 shows that linear estimation tends to overestimate chronological age, particularly in subjects over 18 years of age. Overestimation is strongest for the clavicle. 

Table 3 shows results for age estimation using multiple predictors. Combining the prediction of the clavicle with that of one wisdom teeth considerably improves the prediction: about 14% increase in R² for the upper ones, and about 15% increase for the lower ones. Adding more than one tooth to the prediction does not improve the prediction in any way (all ΔR² < 1%).

## 4. Discussion

The clavicle is a proven biological structure to verify in particular the age of majority in age determinations, particularly when distinguishing between individuals below and above the age of 21 [47]. Some variance in staging is attributed to the raters as well as the imaging modalities, and also the socioeconomic status of the subjects, as described in the literature [48]. In our study, we observed that the three raters did not show significant differences in their findings, despite the variability in their clinical professional experience, which ranged from one to ten years. This highlights the reliability and consistency of the age estimation methods used in our study. Moreover, our study revealed that age determination based on the clavicle was strongly correlated with biological age, but it tended to overestimate the age of subjects above 18 years. This overestimation should be avoided at all costs, as it does not comply with the AGFAD standard. Therefore, age estimation methods that show overestimation beyond the acceptable range should be avoided in forensic applications to prevent erroneous conclusions. In this context, a possible outcome of overestimation in age diagnosis could entail the application of adult criminal law and consequently subject the defendant to a higher sentence. It is worth noting that the applicability of age estimation methods, including those used in our study, is constantly being scientifically reappraised in different populations. Moreover, their efficacy is continuously verified by application studies to evaluate their accuracy, reliability, and validity. This ensures that age estimation methods are based on current scientific knowledge and are effective in forensic and clinical applications [49,50,51]. Cut-off values were developed, which determine a certain stage as a measure for reaching an assignable age limit. In a study by Shedge et al. from India, stage 3a was found to be the reference stage for reaching the age of 18 in 350 subjects (147 females and 203 males) [52]. In contrast, the study by Kellinghaus et al. observed stage 3a for the first time in subjects aged 16.5 (males) and 15.6 (females) [15]. Stage 2c was associated with the minimum age of 16 in the study by Shedge et al. In the recommendation by Schmeling et al., thin-slice CT in Germany uses a cut-off at stage 3c to indicate the completion of 19 years of age [5,52]. Consequently, a review by Hermetet et al. summarizes that the completion of stage 4 can safely be associated with the age of majority at 18 years of age [53]. In the literature related to age determination, it is calculated how important population-related reference tables are in the application in order to achieve a more exact approximation of biological age (source). Tooth stages differ in different populations at the same age [41,54,55]. In contrast, two reviews by Rolseth et al. indicate that there is no conclusive statistical certainty in the population-based differentiation of stages because of age mimicry and a heterogenous study design that, consequently, means they are not comparable with each other [56,57]. The underlying study design is based on a patient collective that underwent a therapeutic measure at the university medical center during the specified period and had an assured age. All subjects were insured in Germany and resided there at the time of the study. As a limitation of the study, our population sample size of 161 subjects as well as the disproportionate share of male subjects (120) should be pointed out, although respective expert opinions concerning unaccompanied male refugees within and outside of criminal proceedings can be found in the literature [17]. This could have an impact on the overall significance of the results and could be evaluated in subsequent future studies.

Of particular interest in our study is the simultaneous presence of wisdom teeth and clavicles, which have otherwise been studied separately in the literature and in different cross-sectional studies as well as in deceased individuals. The simultaneous presence of both features (wisdom teeth and clavicles) for age determination in an individual with known biological age, allowed us to investigate the best combination for approximating age. Here, the combination of the clavicle and a wisdom tooth led to the best result. The wisdom tooth used, which tends to be preferred here, should be either tooth 38 or 48. This was already shown in a previous study by the working group [42] and is consistent with other literature [58]. A study by Bassed et al. also demonstrated the importance of clavicle and wisdom tooth cooccurrence for age determination in a population study of 600 deceased individuals in Australia [20]. In this study set-up as well as in ours, CT images were evaluated, which according to the literature were partly associated with a higher accuracy of findings, especially when it came to the accessibility of impacted wisdom teeth, which would not be equally possible in 2D X-rays [59,60]. Furthermore, the use of three-dimensional imaging techniques, such as CT scans, for age determination in wisdom teeth has raised concerns regarding radiation exposure. The AGFAD standard emphasizes the importance of minimizing unnecessary radiation exposure in forensic investigations [5]. Although CT scans offer detailed and accurate visualization of dental structures, the potential risks associated with radiation exposure should not be overlooked. On the contrary, the literature states that findings based on CT and OPTG radiographs do not show significant differences but would lead to divergent therapeutic approaches in clinical practice [61]. In addition to radiographic techniques, advancements in non-invasive imaging methods, such as magnetic resonance imaging (MRI), have shown potential in assessing dental and skeletal development [62]. MRI does not involve ionizing radiation and can provide detailed images of soft tissues, making it suitable for evaluating dental and skeletal maturation [63]. However, despite the availability of various imaging modalities, it is important to consider their accessibility, cost-effectiveness, and applicability in different forensic settings. In this context, it is crucial for forensic experts to weigh up the risks and benefits of using CT imaging in age estimation and take appropriate measures to minimize the potential adverse effects of radiation exposure. All in all, it can be concluded that the three-dimensional mode of diagnosis of the wisdom teeth is not state of the art because the avoidance of unnecessary radiation exposure must be observed according to the AGFAD standard, and it is not a prerequisite for sufficient diagnosis for the purpose of age diagnosis in wisdom teeth. The reporting mode selected here is based on the presence of both features in a CT image.

## 5. Conclusions

The accuracy of forensic age estimation can be significantly increased when combining data from clavicular and wisdom teeth analyses. 

Furthermore, dental age determination was not relevantly influenced by a rater’s experience: even after a short training exercise, otherwise inexperienced readers reached conclusions similar to those reached by an experienced reader. Within the limitations of this study, the following conclusions could be drawn:

(1) The following hypothesis can be rejected: the dental age determination of the three raters differs according to their expertise. No significant difference could be found between the three raters.
(2) The following hypothesis can only partially be supported: forensic age estimation is more accurate when all five indicators (wisdom teeth 18, 28, 38, 48 and the clavicle) are used. The best results were found when two features were combined (e.g., clavicle and one lower tooth); nevertheless, adding further teeth did not lead to any improvement.

As a result of this study, we would recommend a preferred use of the lower wisdom teeth for dental age estimation in combination with the clavicle.

The present sample is solely based on subjects presently living in Germany and most of them grew up here. Compared to refugees, nutrition in Germany may be better than in some poorer countries, and development of clavicula as well as mineralization of teeth may be influenced. These population differences in the timing of tooth and skeletal development can have implications for age estimation. It is important to consider these population differences when using age estimation methods, and to use appropriate reference standards that are applicable to the population being studied. Hence, the study should be repeated and include subjects from developing countries.

## Figures and Tables

**Table 1:** Sample description.

Variable	Valid Obs.	Missing Obs.	Mean	SD	ICC
Age chronological	161	0	17.59	3.03	-
Age estim. by Clavicula	161	0	18.59	5.08	0.82
Age estim. by Tooth	-	-	-	-	-
18	143	18	19.01	2.75	0.88
28	146	15	19.09	2.75	0.88
38	135	26	19.34	2.89	0.88
48	132	29	19.50	2.80	0.86

Note: There were 41 females and 120 males in the sample.

**Table 2:** Linear prediction of age when utilizing mineralization of each tooth individually.

Variable	Beta	E_beta_	Cons.	R^2^
Clavicula	0.45	0.02	9.19	0.57
Tooth 18	0.87	0.03	0.96	0.62
Tooth 28	0.85	0.03	1.37	0.59
Tooth 38	0.82	0.03	1.44	0.65
Tooth 48	0.87	0.03	0.62	0.62

Note: Calculations were performed on samples between *n* = 399–483, mostly 3 times per person.

**Table 3:** Linear prediction of age when utilizing clavicula development stage and mineralization of several teeth combined.

Variable(s) Clavicula Plus					Beta					SE_beta_					Cons.	ΔR^2^
nothing					0.45					0.02					9.19	0.57
Tooth	18				0.27	0.53				0.02	0.04				2.54	0.14
Tooth		28			0.27		0.53			0.02		0.04			2.46	0.14
Tooth			38		0.26			0.52		0.02			0.03		2.65	0.15
Tooth				48	0.27				0.52	0.02				0.03	2.43	0.15
Teeth			38	48	0.26			0.3	0.25	0.02			0.09	0.09	2.23	<0.01
Teeth		28	38	48	0.24		0.23	0.2	0.18	0.02		0.06	0.09	0.09	1.51	<0.01
Teeth	18	28	38	48	0.23	0.15	0.12	0.17	0.17	0.02	0.08	0.08	0.1	0.09	1.39	<0.01

Note: Calculations were performed on 100 imputed samples with *n* = 161, with three estimates per person. ΔR^2^ compares to 0 in line 1, to 0.57 in lines 2–5, and to the above in lines 6–8.

## Data Availability

The data from this study were part of the dissertation paper of I.F. Data are shown in Figure 1, Figure 2 and Figure 3. Data sets are available upon request.
